# A Trypsin Inhibitor from *Moringa oleifera* Flowers Modulates the Immune Response In Vitro of *Trypanosoma cruzi*-Infected Human Cells

**DOI:** 10.3390/antibiotics9080515

**Published:** 2020-08-14

**Authors:** Isabella Coimbra Vila Nova, Leyllane Rafael Moreira, Diego José Lira Torres, Kamila Kássia dos Santos Oliveira, Leydianne Leite de Siqueira Patriota, Luana Cassandra Breitenbach Barroso Coelho, Patrícia Maria Guedes Paiva, Thiago Henrique Napoleão, Virgínia Maria Barros de Lorena, Emmanuel Viana Pontual

**Affiliations:** 1 Departamento de Bioquímica, Centro de Biociências, Universidade Federal de Pernambuco, Recife 50670-901, Pernambuco, Brazil; isabella.coimbra@ufpe.br (I.C.V.N.); leydianne.patriota@ufpe.br (L.L.d.S.P.); luana.coelho@ufpe.br (L.C.B.B.C.); patricia.paiva@ufpe.br (P.M.G.P.); thiago.napoleao@ufpe.br (T.H.N.); 2 Departamento de Imunologia, Centro de Pesquisas Aggeu Magalhães, Fundação Oswaldo Cruz, Recife 50670-901, Brazil; leyllanemoreira@gmail.com (L.R.M.); diegolira18ufpe@gmail.com (D.J.L.T.); kamilakassia@outlook.com (K.K.d.S.O.); lorena@cpqam.fiocruz.br (V.M.B.d.L.); 3 Departamento de Morfologia e Fisiologia Animal, Universidade Federal Rural de Pernambuco, Recife 52171-900, Brazil

**Keywords:** cytokines, cytotoxicity, immunomodulatory agent, *Moringa oleifera*, protease inhibitor, trypanocidal agent

## Abstract

*Trypanosoma cruzi* causes the lethal Chagas disease, which is endemic in Latin America. Flowers of *Moringa oleifera* (Moringaceae) express a trypsin inhibitor (MoFTI) whose toxicity to *T. cruzi* trypomastigotes was previously reported. Here, we studied the effects of MoFTI on the viability of human peripheral blood mononuclear cells (PBMCs) as well as on the production of cytokines and nitric oxide (NO) by *T. cruzi*-infected PBMCs. Incubation with MoFTI (trypsin inhibitory activity: 62 U/mg) led to lysis of trypomastigotes (LC_50_ of 43.5 µg/mL) but did not affect the viability of PBMCs when tested at concentrations up to 500 µg/mL. A selectivity index > 11.48 was determined. When *T. cruzi*-infected PBMCs were treated with MoFTI (43.5 or 87.0 µg/mL), the release of the pro-inflammatory cytokine TNF-α and INF-γ, as well as of NO, was stimulated. The release of the anti-inflammatory cytokine IL-10 also increased. In conclusion, the toxicity to *T. cruzi* and the production of IL-10 by infected PBMCs treated with MoFTI suggest that this molecule may be able to control parasitemia while regulating the inflammation, preventing the progress of Chagas disease. The data reported here stimulate future investigations concerning the in vivo effects of MoFTI on immune response in Chagas disease.

## 1. Introduction

The Chagas disease, also known as American trypanosomiasis, is an endemic and lethal disease common in Latin America. It is caused by the protozoan *Trypanosoma cruzi* and the transmission occurs when vertebrates come in contact with the feces of infected triatomine insects, popularly known as “kissing bugs”. The current situation of the Chagas disease is a concern, since it is estimated that about 6 to 7 million people worldwide are infected with *T. cruzi* [1].

The life cycle of *T. cruzi* is complex, comprising several evolutionary forms. The vector harbors epimastigotes and metacyclic trypomastigotes in its gut, while the blood trypomastigotes and the intracellular amastigotes are found in vertebrate hosts. Trypomastigotes and amastigotes represent the main targets for therapies [2,3].

The World Health Organization [1] recommends treating Chagas disease using benznidazole and nifurtimox. Both medicines are effective if given early in the acute phase, but their effectiveness is reduced in the more advanced stages; in addition, there is an increase in the frequency of adverse effects with increasing patient age [4,5]. The most common side effects are rashes, fever, generalized edema, lymphadenopathy, myalgia, arthralgia, gastrointestinal disorders, neutropenia, thrombocytopenic purpura, and peripheral polyneuropathy [6].

This scenario stimulates the search for new trypanocidal agents that are more effective in the chronic phase of Chagas disease and less toxic to hosts [7]. The processes of infection of host cells by *T. cruzi* and the survival of the parasite depend on the activity of important proteases; thus, an imbalance in the activity of these enzymes can cause damages to the parasite [8,9,10]. In this sense, *T. cruzi* enzymes represent interesting targets for studies of new therapeutic approaches [11].

It is well reported that some compounds (called protease inhibitors) are able to interact with protease molecules at specific sites, leading to the reduction or blockage of their activities [12]. The deregulation of proteases is a triggering factor for the onset of various pathologies and recent research has pointed out protease inhibitors as promising pharmacological agents [13,14]. Antioxidant, anti-inflammatory, immunomodulatory, antiviral, antimicrobial and antiparasitic activities of these molecules have been demonstrated [15,16,17,18]. Sangenito et al. [19] reported that inhibitors of HIV aspartyl peptidase affected the integrity of cellular structures of *T. cruzi* trypomastigotes, leading to metabolic disorders.

**Figure 1:**
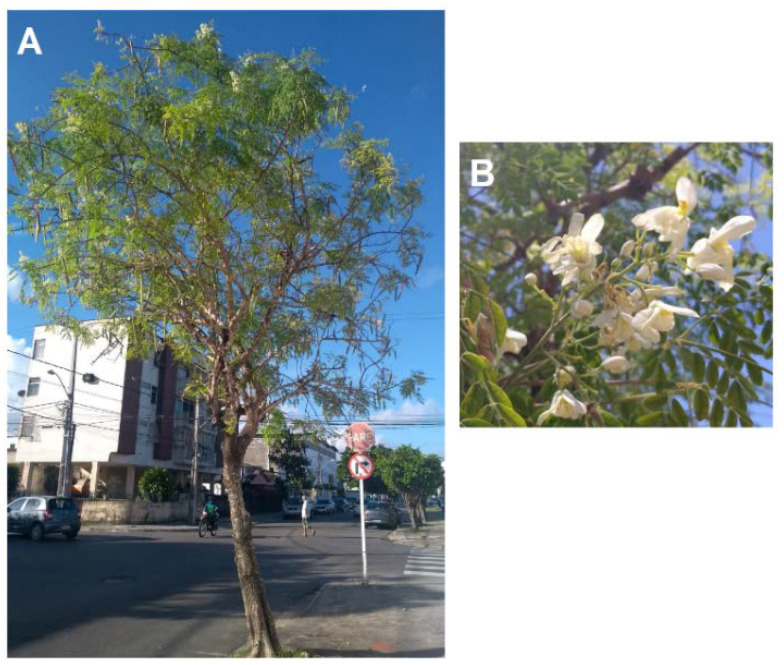
The *Moringa oleifera* tree (**A**) and its flowers (**B**).

*Moringa oleifera* Lamarck (Moringaceae) is a pantropical tree (Figure 1A) that has aroused interest due to its medicinal properties and use as a source of oil and biogas, for example [20,21,22]. Its flowers (Figure 1B) contain a 18-kDa protein called MoFTI with trypsin inhibitory activity and toxicity to *T. cruzi* trypomastigotes with a lethal concentration that led to lysis of 50% of parasites (LC_50_) of 41.20 μg/mL [11]. MoFTI was more toxic to the parasites than to murine macrophages and Vero cells, with selectivity indexes (SI) of 9.8 and >12, respectively [11]. The statement that MoFTI is a trypanocidal agent stimulated the investigations described in the present manuscript. Here, we tested the hypothesis that this inhibitor may interfere with the immune response of human peripheral blood mononuclear cells (PBMCs) infected with *T. cruzi*. In this sense, the assessment of MoFTI effects the viability and production of cytokines and nitric oxide (NO) by infected PBMCs are reported.

## 2. Results and Discussion

**Figure 2:**
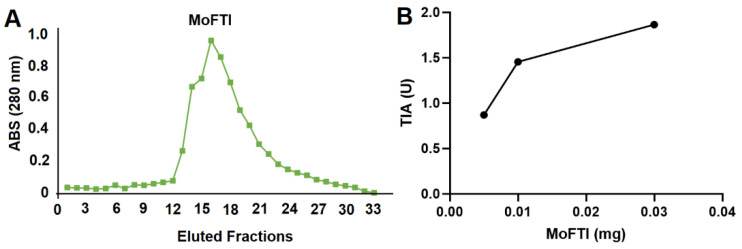
Isolation of MoFTI. (**A**) Affinity chromatography of *M. oleifera* flower extract in Trypsin–Agarose column. The elution step with 0.1 M KCl-HCl pH 2.0 can be seen and fractions of 1.0 mL were collected. (**B**) Trypsin inhibitor activity (TIA) of MoFTI on bovine trypsin.

The affinity chromatography of *M. oleifera* flower extract in the Trypsin–Agarose column resulted in isolation of MoFTI (Figure 2A), which was able to inhibit the hydrolyze of N-α-benzoyl-DL-arginine-p-nitroanilide (BApNA) by trypsin in a dose-dependent way (Figure 2B). The specific trypsin inhibitory activity of MoFTI was 62 U/mg, agreeing with previous reports [11,22]. Pontual et al. [22] reported that MoFTI showed an inhibition constant (Ki) of 2.4 μM on bovine trypsin.

After verifying that the protease inhibitor domain of MoFTI was active, we checked whether its antiparasitic property was also active. It was found that incubation with this inhibitor led to lysis of *T. cruzi* trypomastigotes, since the number of parasite cells counted in treatments with MoFTI was lower than in the negative control. After 24 h, the LC_50_ value was 43.5 (26.2–60.9) μg/mL. Similarly, Pontual et al. [11] reported an LC_50_ value of 41.20 μg/mL for MoFTI on *T. cruzi* trypomastigotes. In this same work, the authors showed that MoFTI was low in toxicity to murine peritoneal macrophages (50% cytotoxic concentration, CC_50_, of 407.01 μg/mL) and did not interfere with the viability of Vero cells at concentrations up to 500 μg/mL.

**Figure 3:**
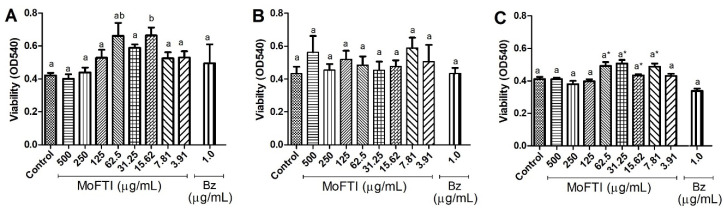
Effects of *M. oleifera* flower trypsin inhibitor (MoFTI) and benznidazole (Bz) on the viability of human peripheral blood mononuclear cells (PBMCs) after exposure by 24 h (**A**), 72 h (**B**), and 120 h (**C**). Different letters (a,b) indicate significant differences between the negative control and the other treatments by analysis of variance (ANOVA) followed by the Kruskal–Wallis test (*p* < 0.05). The asterisk (*) indicates significant differences between Bz and the other treatments. Control: negative control (untreated cells).

In the present work, we investigated the effect of MoFTI on viability of human PBMCs. After 24, 72 and 120 h, it was revealed that MoFTI did not significantly (*p* > 0.05) affect the ability of these cells to metabolize the 3-[4,5-dimethylthiazol-2yl]-diphenyl tetrazolium bromide (MTT), in comparison with negative control (Figure 3). Therefore, it was assumed that, under the conditions used here, MoFTI was not toxic to human PBMCs. As can be seen in Figure 3A, the significantly (*p* < 0.05) greater number of viable cells in treatment with the inhibitor at 15.62 µg/mL, compared to the negative control group, suggest that MoFTI induced cell proliferation after exposure to 24 h. Benznidazole was also not able to interfere with PBMCs viability in comparison with the negative control.

It is well known that molecules with potential for use in therapy of infectious diseases need to be toxic to parasites without affecting the viability of host cells, or at least causing much more damage to parasites than to hosts [23]. The ratio between the CC_50_ value for PBMCs (>500 μg/mL) and LC_50_ for trypomastigotes showed that MoFTI was selective (SI > 11.48) for the parasite regarding these human cells. To the best of our knowledge, this is the first report of MoFTI effects on human cells and this datum suggests that this protein can be an interesting starting material for the production of a new drug for Chagas disease therapy. In fact, it can be expected that a protease inhibitor will end up interacting unwantedly with important enzymes of host cells; however, this is not the only report of a non-cytotoxic protease inhibitor to human cells. For example, the trypsin inhibitor from *Tecoma stans* leaves (TesTI) was also not toxic to human PBMCs [24].

**Figure 4:**
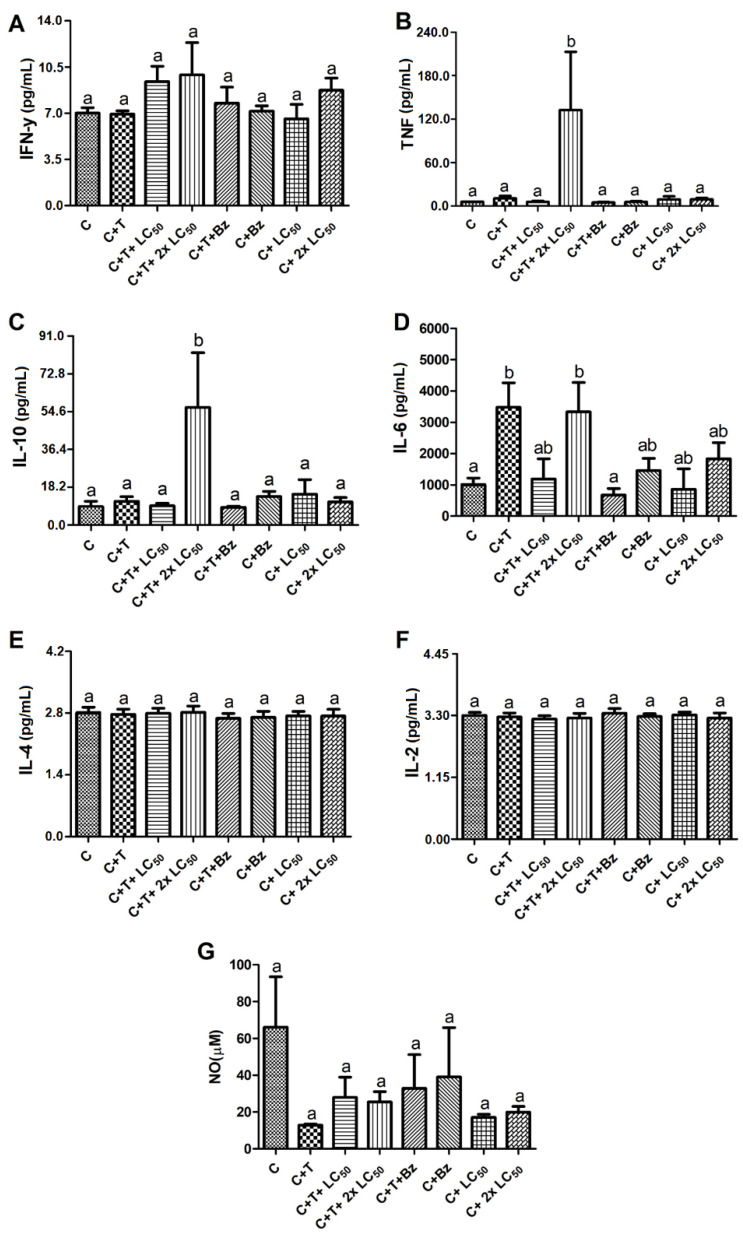
Effect of *M. oleifera* flower trypsin inhibitor (MoFTI) on the production of cytokines (**A**–**F**) and nitric oxide (**G**) by human peripheral blood mononuclear cells (PBMCs) infected or not with *T. cruzi* trypomastigotes after 48-h exposure. The treatments were: negative control (C = untreated and uninfected PBMCs); untreated PBMCs infected with trypomastigotes (C + T); infected PBMCs treated with MoFTI at 43.5 µg/mL (C + T + LC_50_) and 87.0 µg/mL (C + T + 2 × LC_50_); infected PBMCs treated with benznidazole (C + T + Bz); uninfected PBMCs treated with benznidazole (C + Bz); uninfected PBMCs treated with MoFTI at 43.5 µg/mL (C + LC_50_) and 87.0 µg/mL (C+ 2 × LC_50_). Different letters (a,b) indicate significant differences between treatments by analysis of variance (ANOVA) followed by the Tukey post-test (*p* < 0.05).

**Figure 5:**
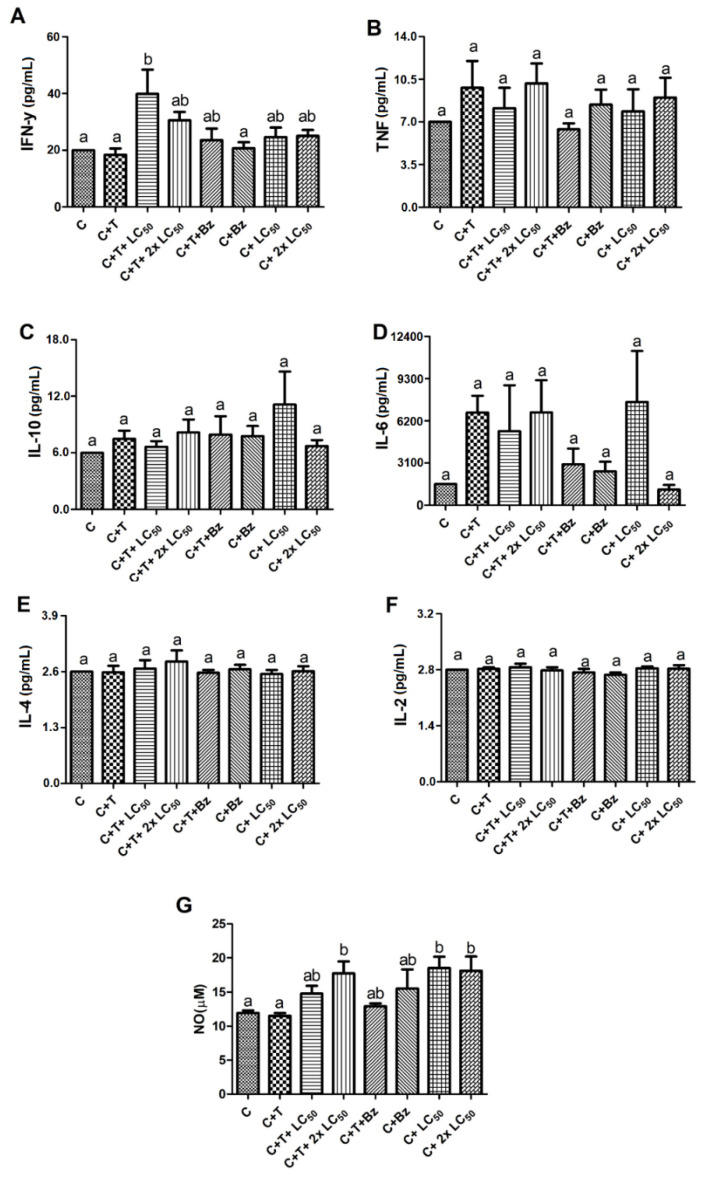
Effect of *M. oleifera* flower trypsin inhibitor (MoFTI) on the production of cytokines (**A**–**F**) and nitric oxide (**G**) by human peripheral blood mononuclear cells (PBMCs) infected or not with *T. cruzi* trypomastigotes after 120-h exposure. The treatments were: negative control (C = untreated and uninfected PBMCs); untreated PBMCs infected with trypomastigotes (C + T); infected PBMCs treated with MoFTI at 43.5 µg/mL (C + T + LC_50_) and 87.0 µg/mL (C + T + 2 × LC_50_); infected PBMCs treated with benznidazole (C + T + Bz); uninfected PBMCs treated with benznidazole (C + Bz); uninfected PBMCs treated with MoFTI at 43.5 µg/mL (C + LC_50_) and 87.0 µg/mL (C+ 2 × LC_50_). Different letters (a,b) indicate significant differences between treatments by analysis of variance (ANOVA) followed by the Tukey post-test (*p* < 0.05).

Previous reports have shown that plant compounds can act as immunomodulatory agents, and this can be interesting from a therapeutic point of view since, when these agents modulate the production of cytokines and other immune mediators, they can increase body defense against pathogens or pathological conditions, even when there is no direct toxicity to the causative agent [25]; because of this, we evaluated the effect of MoFTI on the release of cytokines by PBMCs uninfected or infected by *T. cruzi*. Interestingly, no alterations of cytokine production were observed regarding to negative control group when uninfected PBMCs were exposed to MoFTI (Figure 4 and Figure 5). After 48-h incubation, MoFTI was not able to affect the release of interferon (IFN) γ by infected cells (Figure 4A). On the other hand, the release of tumor necrosis factor (TNF) α and interleukin (IL) 10 (Figure 4B,C, respectively) by *T. cruzi*-infected PBMCs was stimulated by MoFTI at 87.0 µg/mL (2 × LC_50_) in comparison with the control group.

TNF-α is a cytotoxic factor associated with Th1 response against microorganisms, while IL-10 is a Th2 anti-inflammatory interleukin that inhibits the release of pro-inflammatory cytokines. Clinical studies showed that individuals with the chronic cardiac Chagas disease produce pro-inflammatory cytokines, such as TNF-α, to control *T. cruzi* infection. Simultaneously, anti-inflammatory cytokines, especially IL-10, are released to prevent damages to the host tissues and to slow the progress of cardiac complications [26,27]. Individuals at the chronic phase of the indeterminate form of Chagas disease produce higher levels of IL-10 and this is the reason why they do not progress to the clinical stage of Chagas cardiomyopathy [28]. In this sense, the trypanocidal activity of MoFTI, along with the simultaneous stimulation of TNF-α and IL-10 release, suggests that this protein may be able to control parasitemia while regulating the inflammation, preventing the progress of Chagas disease.

Still, after 48-h exposure, increased release of IL-6 was detected for PBMCs infected with *T. cruzi* and treated with MoFTI (2 × LC_50_) at a similar level to that shown by the untreated infected cells (Figure 4D). This datum suggests that the release of IL-6 occurred because of *T. cruzi* infection and not due to MoFTI activity. IL-6 is a pleiotropic cytokine that influences antigen-specific immune responses and inflammatory reactions [29,30]. The profile of the other cytokines investigated here did not change in the untreated infected cells. The release of IL-4, IL-2 and NO by infected cells was not significantly affected by MoFTI after 48-h exposure (Figure 4E–G, respectively).

Unlike the results obtained for 48 h, the infected PBMCs had the release of INF-γ strongly increased in response to treatment for 120 h with both concentrations of MoFTI (Figure 5A) regarding the control. However, no differences were found for the release of TNF, IL-10, IL-6, IL-4 and IL-2 between the treatment groups (Figure 5B–F). When the infected PBMCs were treated with MoFTI at both concentrations, a significant increase in NO release was recorded (Figure 5G), but this does not seem to depend on the infection, because a similar result was detected for uninfected PBMCs treated with the inhibitor. The NO release may have occurred due to direct action of MoFTI or in response to the production of INF-γ [31]. Benznidazole did not affect cytokine release by both uninfected and infected cells.

NO plays an important role in the defense of macrophages against *T. cruzi* by damaging parasite biochemistry and causing, for example, the inhibition of metalloproteins that mediate crucial metabolic processes, including cruzipain. This enzyme participates in parasite nutrition and the infection of host cells [32].

## 3. Materials and Methods

### 3.1. Isolation of MoFTI

The collection of *M. oleifera* flowers occurred in Recife city (8°02′57.9″ S, 34°56′47.8″ W), Pernambuco, Brazil, and a voucher specimen (number 73345) is deposited at the herbarium *Dárdano de Andrade Lima* (*Instituto Agronômico de Pernambuco*, Recife, Brazil). MoFTI was purified as described by Pontual et al. [22]. The procedure started from the maceration (10 min at 27 °C) of fresh flowers (50 g) with distilled water (100 mL) in a blender. The mixture was filtered through gauze and centrifuged (9000× *g*, 15 min, 4 °C) to remove suspended material. The crude preparation was dialyzed (6 h) against 0.1 M Tris-HCl, pH 8.0, containing 0.02 M CaCl_2_ and loaded (4 mL; 66.8 mg of proteins) onto a Trypsin–Agarose (Sigma-Aldrich, St. Louis, MO, USA) column (4.5 × 1.0 cm). MoFTI was eluted with 0.1 M KCl-HCl, pH 2.0, and the presence of proteins in the collected fractions was accompanied by the measurement of absorbance at 280 nm. After dialysis (16 h) against distilled water, MoFTI was lyophilized to dryness and resuspended to a concentration of 1000 μg/mL in distilled water, for assessment of trypsin inhibitory activity, or in Rockwell Park Memorial Institute 1640 (RPMI 1640) complete medium (Sigma-Aldrich), for the assays with *T. cruzi* or PBMCs. Protein concentration was determined according to Lowry et al. [33] using bovine serum albumin (31.25–500 μg/mL) as standard.

### 3.2. Trypsin Inhibitory Activity

The ability of MoFTI to inhibit trypsin was assayed according to Pontual et al. [22]. In a microtiter plate, bovine trypsin (5 µL, 0.1 mg/mL in 0.1 M Tris-HCl, pH 8.0, containing 0.02 M CaCl_2_) was added to 5 µL of 8.0 mM BApNA and MoFTI (0.005 to 0.03 mg/mL). Next, each well received Tris buffer to complete a volume of 200 µL. After incubation (30 min at 37 °C), the absorbance at 405 nm was measured using a microplate reader (Multiskan, Thermo Fisher Scientific, Waltham, MA, USA). One unit of trypsin inhibitor activity corresponded to the amount of MoFTI able to reduce the absorbance by 0.01 in accordance with trypsin activity in absence of the inhibitor.

### 3.3. Obtaining of T. cruzi Trypomastigotes

Cryopreserved trypomastigotes (10^7^ trypomastigotes/mL) were thawed in a water bath at 37 °C. After centrifugation (400× *g*, 10 min, 22° C), the supernatant was discarded, and the pellet resuspended with 5 mL of complete Roswell Park Memorial Institute (RPMI) 1640 medium. The suspension was distributed in culture bottles containing Vero cells and incubated (37 °C, 5% CO_2_) for 24 h. Next, the supernatant was removed to withdraw parasites that did not infect cells. RPMI 1640 complete medium (5 mL) was added and the cultures were incubated for 7 days. During this time, the multiplication of intracellular parasites was daily observed using an inverted microscope. After cell disruption, the trypomastigotes were collected for the subsequent assays.

### 3.4. Trypanocidal Activity of MoFTI

Culture-derived trypomastigotes (Y strain) were maintained by weekly passages in Vero cells cultured in complete RPMI 1640 medium. Trypomastigotes (10^6^ parasites/mL, 100 μL) were placed in 96-well plates and treated with MoFTI (100 μL, 6.25–100 μg/mL) in RPMI medium supplemented with 10% fetal bovine serum (FBS). In negative control, parasites were incubated with complete RPMI medium in the absence of MoFTI. Benznidazole (1.0 µg/mL) was used as positive control. The assays were incubated for 24 h at 37 °C. Next, the number of trypomastigotes was determined by counting cells using a Neubauer chamber in a E100 LED microscope (Nikon, Melville, NY, USA). The percentage of lysis (%) was estimated regarding the number of trypomastigotes in the negative control (100%). Three independent experiments were performed in quadruplicate and the concentration (µg/mL) of MoFTI that leads to lysis of 50% of trypomastigote cells (LC_50_) for 24 h was calculated using the software MedCalc version 17.9.7 (MedCalc Software bvba, Ostend, Belgium).

### 3.5. Isolation of Human PBMCs

The blood collection was performed following the protocol approved (process: 07511612.2.0000.5190) by the Research Ethics Committee of the Instituto Aggeu Magalhães/Fundação Oswaldo Cruz (IAM/FIOCRUZ). The blood was collected from ten healthy individuals (6 women and 4 men) through a vacuum system (Vacutainer; BD Biosciences, Franklin Lakes, NJ, USA) in tubes containing sodium heparin, and homogenized (15 mL) with an aliquot of filtered and sterile phosphate buffered saline (PBS), pH 7.2 (15 mL). Next, 15 mL of this mixture was added to 50 mL Falcon polypropylene tubes containing 15 mL of Ficoll-Hypaque (GE Healthcare Life Sciences, Uppsala, Sweden). After centrifugation (900× *g*, 30 min, 20 °C), PBMCs appeared as a ring between Ficoll and plasma and were collected using a sterile transfer pipette and placed in 15 mL Falcon polypropylene tubes. PBMCs were resuspended using 14 mL of incomplete RPMI 1640 medium containing 1% penicillin/streptomycin and centrifuged (400× *g*, 10 min). This procedure was repeated twice. Following, the cells were resuspended in 2 mL of RPMI 1640 complete medium supplemented with FBS (10%) and containing 1% penicillin/streptomycin. The cells (10 µL) were subsequently stained with Trypan Blue (Sigma-Aldrich) dye (90 µL) and counted in a Neubauer chamber. The number of cells were recorded and adjusted to the desired concentration of 10^6^ cells/mL.

### 3.6. Effect of MoFTI on PBMCs Viability

The effect of MoFTI on viability of PBMCs was assayed measuring the activity of mitochondrial succinate dehydrogenase [34]. Cell suspension (10^6^ cells) was placed in 96-well culture plates with complete RPMI 1640 medium and exposed to MoFTI (3.9 to 500.0 µg/mL). In negative control, PBMCs were incubated with RPMI 1640 medium in absence of MoFTI. Benznidazole (1.0 µg/mL) was also tested. The plates were incubated at 37 °C and 5% CO_2_ for 24, 72 and 120 h. Next, the culture medium was removed and RPMI medium containing 5.0 mg/mL of MTT (Sigma-Aldrich) was added. After incubation (37 °C, 5% CO_2_) for 3 h, the culture medium containing MTT was removed and 100 µL of dimethyl sulfoxide was added. The presence of formazan crystals derived from MTT reduction was immediately recorded by measuring the absorbance at 540 nm. The experiment was conducted in triplicates and the selectivity index (SI) was calculated from the ratio between cytotoxicity to PBMCs (CC_50_) and the LC_50_ of MoFTI.

### 3.7. Treatment of T. cruzi-Infected PBMCs with MoFTI

PBMCs (1 mL, 2 × 10^6^ cells) in complete RPMI medium were placed in 48-well polystyrene culture plates, which were incubated (37 °C, 24 h) to fix adherent cells (mainly monocytes). Next, 0.5 mL of the RPMI medium was removed from each well, and trypomastigotes (0.5 mL, 10^6^ cells) were added. The plates were incubated (37 °C, 5% CO_2_) for 2 h to allow the infection of PBMCs. The mixture (RPMI medium containing infected adherent and non-adherent cells) was treated with MoFTI at 43.5 µg/mL (LC_50_) and 87.0 µg/mL (2 × LC_50_). In negative control, the mixture was maintained without MoFTI, while benznidazole (1.0 µg/mL) was used in positive control. To compare the cell response in the presence or absence of parasites, uninfected cells were incubated in the absence or presence of MoFTI (LC_50_ and 2 × LC_50_) or benznidazole (1.0 µg/mL), as described above. The plates were incubated (37 °C, 5% CO_2_) for 48 or 120 h, and 700 µL of the supernatant from each well was removed and immediately stored at −20 °C for later use to measure cytokine and nitric oxide (NO) levels.

### 3.8. Effect of MoFTI on Cytokine Release by T. cruzi-Infected PBMCs

The release of the cytokines IL-2, IL-4, IL-6, IL-10, IFN-γ and TNF-α by PBMCs was quantified using the Cytometric Bead Array (CBA) system, following the instructions of the manufacturer (BD Biosciences). The data were acquired on the FACScalibur flow cytometer. The acquisitions and analyses were performed using the CellQuestPro software (BD Biosciences) and 5000 events were acquired within the lymphocyte population. The analyses were performed using the BD CBA software.

### 3.9. Effect of MoFTI on NO Production by T. cruzi-Infected PBMCs

Aliquots (50 µL) of the supernatants removed from the plates referred to in Section 3.7 were added to the Griess reagent (50 µL) in 96 well microplates, according to Resende et al. [35]. After incubation (28 °C, 15 min), NO production was estimated by measuring the absorbance at 540 nm and a standard curve of nitrite (3.12–400.0 µM).

### 3.10. Statistical Analysis

The results regarding cytokine and NO levels were statistically evaluated using the GraphPad Prism 5.0 software (GraphPad Software Inc, San Diego, CA, USA). The data that passed the Kolmogorov–Smirnov normality test were analyzed using analysis of variance (ANOVA) followed by the Tukey post-test, while the data that did not pass the normality test were analyzed by the Kruskal–Wallis test followed by the Dunnet post-test. Significance level at 5% was considered.

## 4. Conclusions

The data reported herein point to the trypanocidal trypsin inhibitor of *M. oleifera* flowers as an immunomodulatory agent on *T. cruzi*-infected human PBMCs by stimulating the release of pro-inflammatory (TNF-α and INF-γ) and anti-inflammatory (IL-10) cytokines, as well as NO. In addition, MoFTI was more toxic to the parasite cells than to the human immune cells. Our findings stimulate future investigations of MoFTI in vivo effects on immune response in Chagas disease. It should be remembered that MoFTI is a proteinaceous inhibitor. In this sense, some points must be addressed to develop drug formulations using MoFTI, such as its stability and immunogenicity. Finally, strategies for producing MoFTI at a large scale must be designed so that it can be inserted in the pharmaceutical industry.
